# The current status of chronic kidney disease-mineral and bone disorder management in China

**DOI:** 10.1038/s41598-022-20790-8

**Published:** 2022-10-06

**Authors:** Ya Zhan, Xin He, Daqing Hong, Li Wang, Guisen Li

**Affiliations:** Renal Department and Nephrology Institute, Sichuan Provincial People’s Hospital, School of Medicine, University of Electronic Science and Technology of China, No. 32, West 2nd Duan, 1st Circle Road, Qingyang District, Chengdu, 610072 Sichuan People’s Republic of China

**Keywords:** Evolution, Bone development

## Abstract

Chronic kidney disease-mineral and bone disorder (CKD-MBD) is one of the most common complications of patients with chronic kidney disease (CKD). After considering updated international and domestic CKD-MBD management guidelines, the current management status of CKD-MBD in China remains unclear. We aimed to investigate the management status of CKD-MBD in China. A nationwide survey was combined with a real-world study from Sichuan. Targets recommended in KDIGO 2017 and Chinese 2019 guidelines were used as targets. We compared the data between Sichuan from western China and the eastern developed areas of China, and also compared the results of the real-world data from Sichuan with those of DOPPS5. In the questionnaire, a total of 51,039 maintenance hemodialysis (MHD) patients from 272 centers were involved. Estimated achievement rates for Ca (2.1–2.5 mmol/L), P (1.13–1.78 mmol/L), and iPTH (150–600 pg/mL) levels were 57.1%, 41.1% and 52.0%, respectively. Differences in MBD management between Sichuan from questionnaire and central region. In the real-world survey, a total of 7,053 patients were enrolled. Among them, 57.6%, 24.3%, and 55.0% of patients met corrected Ca, serum P, and iPTH targets specified in Chinese 2019 guidelines, respectively. The comprehensive achievement rate was 7.5%. There are differences in MBD management between Sichuan and DOPPS5 regions, with Sichuan being relatively poorer. The level of the above parameters varies among different genders, age groups, and hospital grades. The achievement rate of serum P was higher in tertiary hospitals and elderly patients (*P* < 0.05). Current MBD management is poor. Phosphate levels in patients treated in secondary and lower hospitals and young dialysis patients should be strengthened.

## Introduction

Chronic kidney disease (CKD) is a global health problem characterized by its high incidence, high mortality rate, and multiple complications. The prevalence of CKD has been estimated as 9.1%, with the total number of CKD patients reaching about 700 million globally, and 130 million in China^1^. The of CKD patients is much shorter than that of the general population. A cohort study consisting of 506,849 Chinese adults demonstrated that severe CKD increased mortality risk, reduced life expectancy, and increased medical costs^2^. In particular, cardiovascular mortality was increased in patients starting dialysis *vs.* the general population^3^.

A variety of complications contribute to adverse clinical outcomes. Among these, CKD-mineral and bone disorder (CKD-MBD) is one of the most important complications. CKD-MBD is a clinical syndrome that includes a series of laboratory abnormalities, vascular or soft tissue calcification, as well as bone diseases^4,5^. Results derived from Dialysis Outcomes and Practice Patterns Study (DOPPS), CORES, and COSMOS indicated that calcium (Ca), phosphorus (P), and intact parathyroid hormone (iPTH) disorders increase the risk of all-cause and cardiovascular mortality in maintenance hemodialysis (MHD) patients^6–8^. Correcting abnormal Ca, P, and iPTH has the potential to significantly improve survival and alleviate the financial burdens of patients with MHD. In particular, consistently meeting the targets of Ca, P, and iPTH recommended by the guidelines can significantly improve the outcomes and decrease the mortality of MHD patients^9^. If one target is not met, mortality risk would increase by 15–21%, and mortality risk in patients who fail to meet all three targets increased by 51%^9^.

Despite updates to international and domestic CKD-MBD management guidelines, the current management status of CKD-MBD in China remains unclear. The latest DOPPS 5 Reports on China were limited to Beijing, Guangzhou, and Shanghai; they failed to represent the management status of China. Therefore, the study combined a questionnaire survey of dialysis centers across the country with a real-world investigation in Sichuan to improve our understanding of the current status of CKD-MBD management in China and provide basic information for future prevention and treatment.

## Methods

### Questionnaire

An online questionnaire was completed by physicians responsible for MHD patient management. CKD patients on hemodialysis (HD) for ≥ 3 months were enrolled from April to July 2019. Patient numbers in each center, average dialysis vintage, the estimated achievement rate for serum Ca, P, and iPTH, and the estimated proportion of patients taking medications for iPTH and P levels management were collected. We compared the results between Sichuan Province from western China and the eastern developed areas while calculating the estimated achievement rate.

### Real-world survey

We conducted a real-world study in Sichuan Province, which completed the most questionnaires**.** Data were extracted from the Medical Quality Information Management System of Kidney Diseases in Sichuan Province. ID number, gender, age, Ca, P, iPTH, hemoglobin, and albumin levels were collected. MHD patients who were registered in the system from July 1, 2020, to June 30, 2021; underwent at least one Ca, P, or iPTH testing; and were > 18 years old, were included. All individuals who had extremely abnormal values (iPTH < 1 pg/mL, P < 0.1 mmol/L or P > 8 mmol/L, and Ca > 5 mmol/L) or missing values were excluded. For each patient, the last pre-dialysis data collected during the study period were assessed. Corrected Ca was calculated as serum Ca (mmol/L) + 0.02 × (40-serum albumin)^5^. Serum P and Ca were measured by spectrophotometry assay. iPTH was measured by immunochemiluminometric assays.

The study was approved by the Ethics Committee of Sichuan Provincial People’s Hospital (No.2021-514-1). All patients signed the written informed consent, and all methods were performed in accordance with the relevant guidelines and regulations, and all methods were performed in accordance with the relevant guidelines and regulations.

### Targets for assessing the achievement rate

Guidelines of 2017 Update by KDIGO^10^ recommended that the corrected serum Ca, serum P, and iPTH should be 2.10–2.54 mmol/L, 0.81–1.45 mmol/L, and 150–600 pg/mL, respectively. In 2019, China CKD-MBD guidelines^5^ recommended that targets for each MBD parameter: corrected serum Ca, 2.1–2.5 mmol/L; serum P, 0.87–1.45 mmol/L; and iPTH 2–9 times (150–600 pg/mL) of the upper limit of the normal range. Values of *P* < 0.87 mmol/L and > 1.45 mmol/L were defined as hypophosphatemia and hyperphosphatemia, respectively.

### Statistics

The total estimated achievement rates for Ca, P, and iPTH were weighted based on achievement rates and patient numbers reported by each center. In China, hospitals are grouped into different grades, including primary, secondary and tertiary. Tertiary hospitals include grade III A and grade III B hospitals. Secondary and lower hospitals refer to secondary and primary hospitals. At present, China is divided into four major economic regions, namely, the eastern region, the central region, the western region, and the northeastern region. According to China's Gross Domestic Product (GDP) in recent years, we ranked the economic conditions between the four major economic regions by GDP and were divided into super high GDP, high GDP, moderate GDP, and low GDP regions. Since the contents were descriptive, we did not use any method to fill in missing data. All the statistical analyses were performed using SPSS and Microsoft Excel. A *P*-value less than 0.05 was considered statistically significant.

### Statement of Ethics

This case report was approved by the Ethics Committee of Sichuan Provincial People’s Hospital (No. 2021-514-1).

## Results

### Nationwide CKD-MBD questionnaire survey

#### Achievement rate for each target

**Figure 1:**
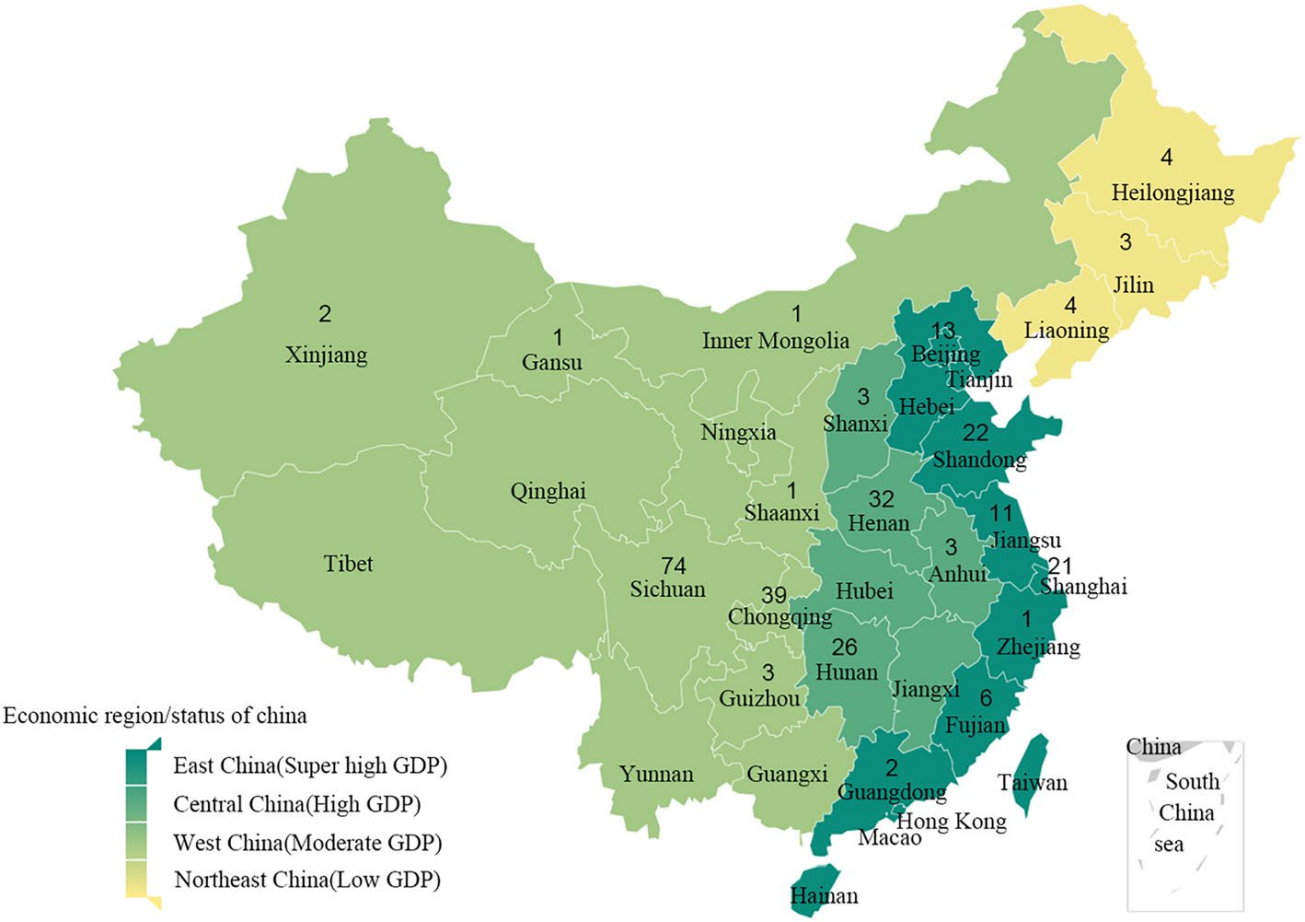
Economic regions distribution of included dialysis centers in China. *GDP* Gross domestic product.

In total, 272 dialysis centers (Fig. 1) covering 51,039 MHD patients within 21 provinces in China responded to the online survey. Of these centers, the number of patients with HD varied from 6 to 848, with a median value of 164 (94,240). A dialysis vintage < 3 years, 3–5 years, 5–7 years, 7–10 years, and 10–15 years accounted for 1.7%, 30.3%, 43.7%, 18.7%, and 5.6% of patients, respectively.

**Figure 2:**
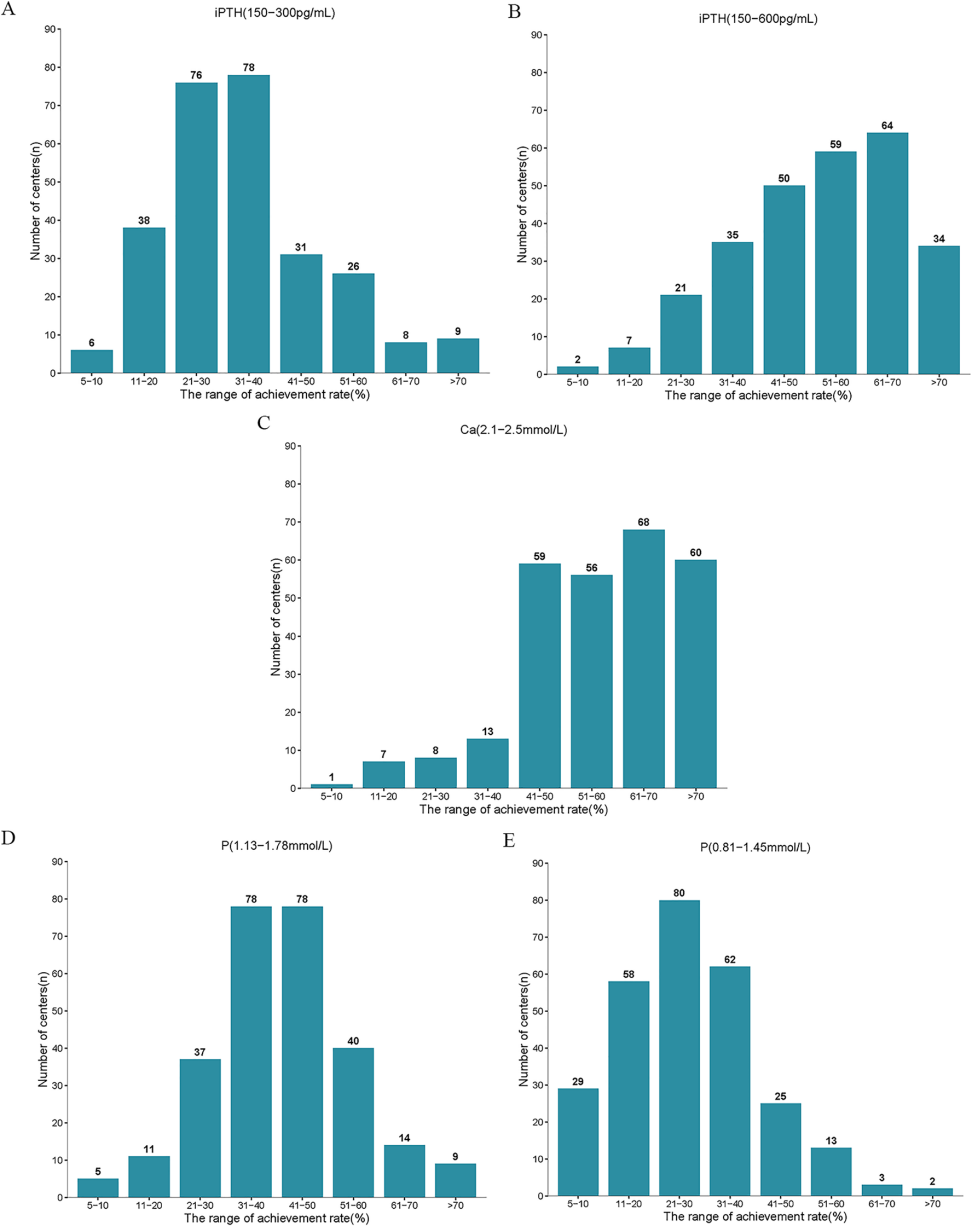
Distributions of iPTH, Ca, and P achievement rates in the included centers. Ca, Calcium; P, Phosphorus; iPTH, Intact parathyroid hormone.

While taking 150–300 pg/mL as a treatment target for iPTH, the achievement rate ranged from 21 to 40% (Fig. 2A) and the estimated overall achievement rate was only 33.8%. When considering 150–600 pg/mL as the target, the achievement rate ranged from 41 to 70% (Fig. 2B) and the estimated achievement rate increased to 52.0%. In addition, 13.6% (37/272) of these centers measured iPTH every 6 months, and 11.4% (31/272) centers did it irregularly.

89.3% (243/272) of centers had an achievement rate of more than 40% for the target of serum Ca (2.1–2.5 mmol/L) (Fig. 2C), and the estimated achievement rate was 57.1%. There were 36.0% (98/272), 87.9% (239/272) and 9.6% (26/272) centers which routinely used 1.25 mmol/L,1.5 mmol/L and 1.75 mmol/L dialysate Ca concentrations, respectively.

For the target of 1.13–1.78 mmol/L for phosphate, 57.4% (156/272) of centers had an achievement rate of 31–50% (Fig. 2D) and an estimated 41.1% of patients achieved target values. After restricting the target range to 0.81–1.45 mmol/L, 61.4% (167/272) of centers had an achievement rate of < 30% (Fig. 2E) and the achievement rate dropped to 26.7%.

#### Comparison of achievement rate between the eastern region and Sichuan

The questionnaire was completed by 74 and 76 centers in Sichuan and the eastern region of China, respectively. When the iPTH target was set at 150–300 pg/mL, the estimated achievement rate in Sichuan was slightly lower than that in the east region (33.2% *vs.* 35.1%, *P* = 0.001), and for the target of 150–600 pg/mL, the estimated achievement rate in Sichuan was still lower than that in the eastern region (52.0% *vs.* 55.2%, *P* < 0.001). Similarly, the estimated achievement rate of serum Ca was lower in Sichuan than in the east region (54.5% *vs.* 60.2%, *P* < 0.001). There was no significant difference in serum P estimated achievement between Sichuan and eastern regions, whether the target was 1.13–1.78 mmol/L (41.0% vs. 40.8%, *P* = 0.761) or 0.81–1.45 mmol/L (25.8% *vs.* 26.2%, *P* = 0.554).

#### Medications used for CKD-MBD management

**Figure 3:**
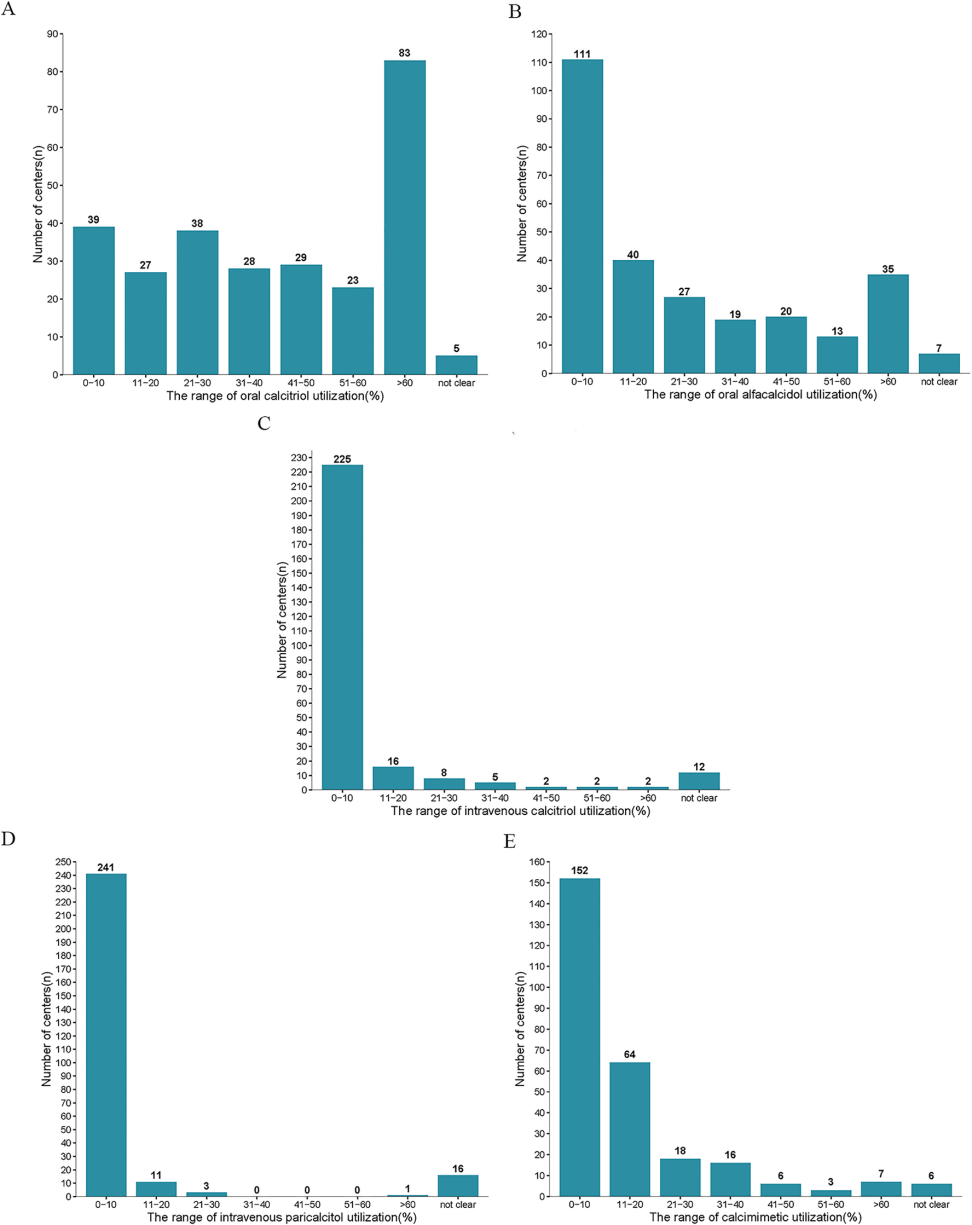
Distribution of utilization rates of different medications used to reduce iPTH levels in the included centers.

The utilization rate of oral calcitriol was > 60% in 30.5% (83/272) centers in China (Fig. 3A). The centers with utilization rates of oral alfacalcidol < 10%, accounted for 40.8% (111/272) centers (Fig. 3B). 82.7% (225/272), 88.6% (241/272) centers and 55.9% (152/272) centers reported a utilization rate less than 10% of intravenous calcitriol, or paricalcitriol, or calcimimetic, respectively (Fig. 3C, D and E).

**Figure 4:**
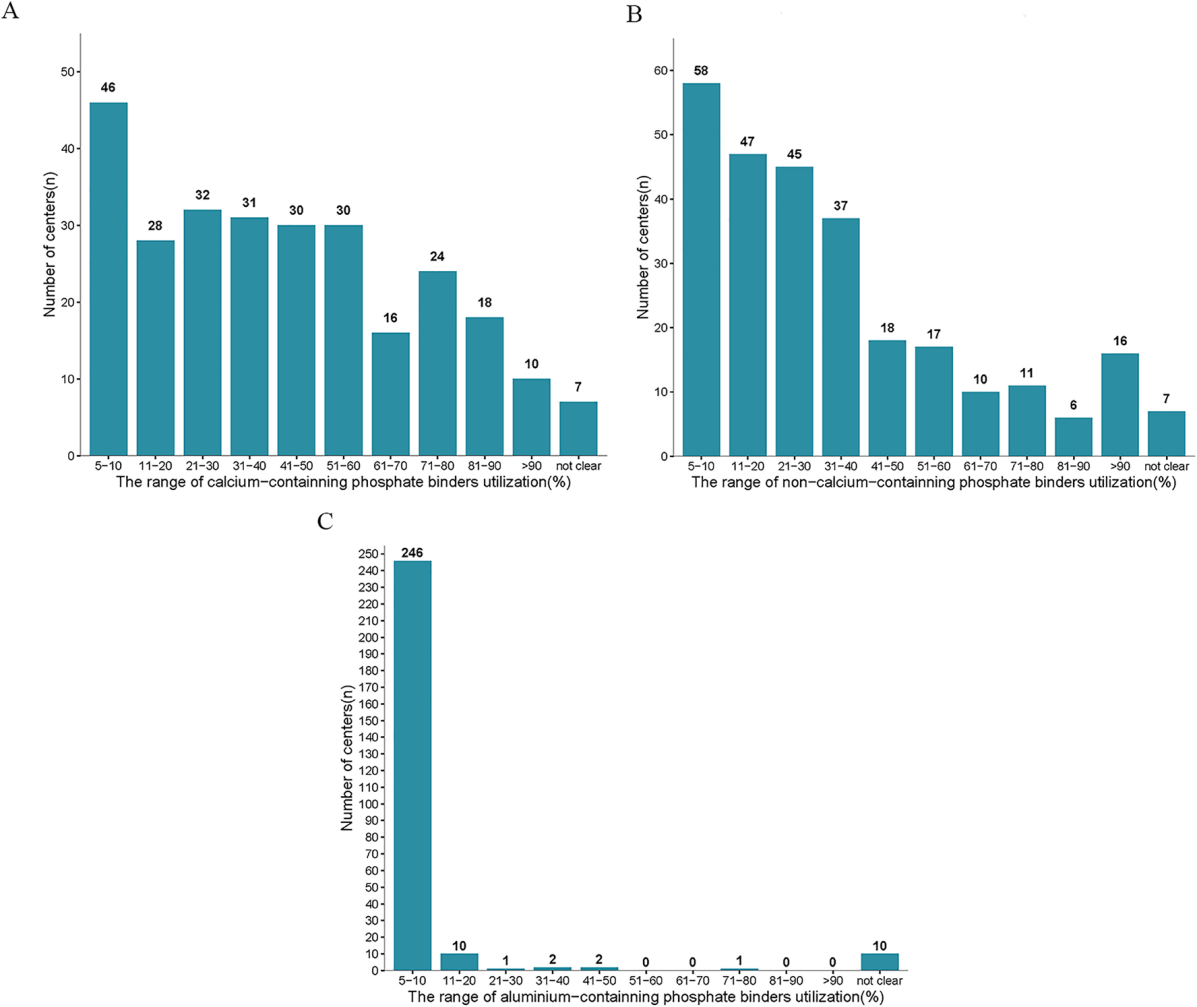
Distribution of utilization rates of different medications used to reduce P levels in the included centers.

In 61.4% (167/272) centers, the utilization rate of Ca-containing phosphate binders was less than 50% (Fig. 4A). The utilization rate of non-Ca-containing phosphate binders in 150 (55.1%) centers was < 30% (Fig. 4B). Aluminum-containing phosphate binder was still used in 90.4% (246/272) of centers although the utilization rate was < 10% (Fig. 4C).

### Real-word survey from Sichuan

#### Achievement rate for each target

Sichuan province was selected, which has the highest number of completed questionnaires, for the real-world study. Data were extracted from our database. Altogether, 7,053 HD patients were included. In total, 4,195 (59.5%) patients were male and 5,099 (72.3%) were treated at tertiary hospitals. The median age of all included patients was 55 (47,67) years. Median corrected Ca, P, and iPTH levels were 2.21 (2.05,2.37) mmol/L, 1.78 (1.40,2.18) mmol/L, and 314.3 (164.0, 560.0) pg/mL, respectively (Table 1).

**Table 1:** The basic clinical information of MHD patients.

	Value
Total patients (n)	7053
Male, n (%)	4195 (59.5%)
Tertiary hospital, n (%)	5099 (72.3%)
Age (year)	55 (47, 67)
Corrected Ca (mmol/L)	2.21 (2.05, 2.37)
Serum P (mmol/L)	1.78 (1.40, 2.18)
iPTH (pg/mL)	314.3 (164.0, 560.0)

According to 2019 China CKD-MBD guidelines, achievement rates of corrected Ca, P, and iPTH values were 57.6%, 24.3%, and 55.0%, respectively. Additionally, when serum P targets were set at 1.13–1.78 and 0.81–1.45 mmol/L, achievement rates were 39.7% and 25.5%, respectively.

#### Comparison of achievement rate between Sichuan and DOPPS5

As shown in Table 2, the achievement rates for corrected Ca (72% *vs.* 77% *vs.* 57.6%, *P* < 0.001) and serum P (54%, 56%, 39.7%, *P* < 0.001) of DOPPS5 data from Europe and North America^11^ were greater than those of Sichuan. iPTH achievement rate of Sichuan was not different significantly from Europe (55.0% *vs.* 57%, *P* = 0.062) while lower than that of North America (55.0% *vs.* 64%, *P* < 0.001). The achievement rate of corrected Ca in Sichuan was lower than that of Chinese DOPPS5^11^ (57.6% *vs.* 62%, *P* < 0.001). There were no between-group differences in the achievement of target serum P (39.7% *vs.* 39%, *P* = 0.669) and iPTH (55.0% *vs.* 52%, *P* = 0.059) levels were observed.

**Table 2:** Comparison of corrected Ca, P, and iPTH achievement rates with DOPPS5.

	Sichuan (n = 7053)	China-DOPPS5 (n = 1186)	Europe (n = 3651)	North america (n = 13,080)	*P*
Corrected Ca, n	5583	1186	3651	13,080	
**Corrected Ca (mmol/L), %**					
< 2.1	31.3^abc^	25	20	12	< 0.001
2.1–2.5	57.6^abc^	62	72	77	< 0.001
> 2.5	11.1^b^	13	8	11	< 0.001
P, n	6413	1186	3651	13,080	
**P (mmol/L), %**					
< 1.13	10.9^ab^	7	17	12	< 0.001
1.13–1.78	39.7^bc^	39	54	56	< 0.001
> 1.78	49.4^abc^	55	29	33	< 0.001
iPTH, n	5610	1186	3651	13,080	
**iPTH (pg/mL), %**					
< 150	21.3^abc^	27	30	18	< 0.001
150–600	55.0^c^	52	57	64	< 0.001
> 600	24.1^bc^	21	14	18	< 0.001

^a^Sichuan compared with China-DOPPS5, *P* < 0.05.

^b^Sichuan compared with Europe, *P* < 0.05.

^c^Sichuan compared with North America, *P* < 0.05.

In total, 4,385 patients had concurrent Ca, P and iPTH results. 15.1% (663/4,385) patients reached both corrected Ca and serum P targets, while 31.5% (1,383/4,385) met standards of both corrected Ca and iPTH. Finally, 12.8% (562/4,385) patients met both iPTH and serum P level target. Only 7.5% (331/4,385) patients had Ca, P, and iPTH levels within the target ranges of all three parameters.

#### Concomitant achievement rate for Ca, P and iPTH

Compared with tertiary hospitals, percentages of patients who met the standards of serum P (17.6% *vs.* 27.1%, *P* < 0.001) and corrected Ca (55.2% *vs.* 58.4%, *P* = 0.039) in secondary and lower hospitals were lower and the prevalence of hyperphosphatemia was higher (80.2% *vs.* 68.1%, *P* < 0.001), especially in patients with serum P levels > 1.78 mmol/L (59.4% *vs.* 45.4%, *P* < 0.001). The percentage of patients with who met the standard of iPTH in tertiary hospitals was lower that of patients in primary hospitals (25.0% *vs.* 28.1%, *P* = 0.017). When the target range for iPTH was defined from 150 to 600 pg/mL, the achievement rate was similar between tertiary and secondary and lower hospitals (55.0% *vs.* 55.2%, *P* = 0.859) (Table 3).

**Table 3:** The levels of Ca, P, and iPTH in different hospital grades.

	All patients (n = 7053)	Tertiary hospitals (n = 5099)	Secondary and lower hospitals (n = 1954)	*P*
Corrected Ca, n	5583	4174	1409	
Corrected Ca (mmol/L)	2.21 (2.05, 2.37)	2.21 (2.06, 2.37)	2.19 (2.04, 2.36)	0.117
**Corrected Ca, n (%)**				
< 2.1	1747 (31.3)	1285 (30.8)	462 (32.8)	0.161
2.1–2.5	3214 (57.6)	2436 (58.4)	778 (55.2)	0.039
> 2.5	622 (11.1)	453 (10.8)	169 (12.0)	0.239
Serum P, n	6413	4569	1844	
Serum P (mmol/L)	1.78 (1.40, 2.18)	1.71 (1.35, 2.11)	1.92 (1.54, 2.34)	< 0.001
**Serum P, n (%)**				
< 0.87	260 (4.1)	220 (4.8)	40 (2.2)	< 0.001
0.87–1.45	1561 (24.3)	1236 (27.1)	325 (17.6)	< 0.001
1.46–1.78	1423 (22.2)	1040 (22.8)	383 (20.8)	0.082
> 1.78	3169 (49.4)	2073 (45.4)	1096 (59.4)	< 0.001
iPTH, n	5610	4087	1523	
iPTH (pg/mL)	314.3 (164.0, 560.0)	323.6 (166.1, 569.7)	291.0 (154.0, 533.0)	< 0.001
**iPTH, n (%)**				
< 150	1249 (22.3)	885 (21.7)	364 (23.9)	0.072
150–300	1448 (25.8)	1020 (25.0)	428 (28.1)	0.017
301–600	1639 (29.2)	1226 (30.0)	413 (27.1)	0.035
601–800	439 (7.8)	325 (8.0)	114 (7.5)	0.563
> 800	835 (14.9)	631 (15.3)	204 (13.4)	0.056

#### Effect of gender and age on the achievement rate

There were no differences in compliance rates of corrected Ca and P (*P* > 0.05) between male and female patients. However, the percentage of male patients with iPTH reaching 150–300 pg/mL was higher than that of female patients (27.2% *vs.* 23.8%, *P* = 0.004). When the target range for iPTH was adjusted to 150–600 pg/mL, the compliance rate of male patients (56.5% *vs.* 52.9%, *P* = 0.009) was also higher than that of female patients. The incidence of hypocalcemia in male patients (34.0% *vs.* 27.3%, *P* < 0.001) was significantly increased than that of female subjects, and the percentage of patients with hypercalcemia was reduced significantly in male *vs.* female subjects (9.0 *vs.* 14.3%, *P* < 0.001) (Table 4).

**Table 4:** The levels of Ca, P, and iPTH in different-gender groups.

	All patients (n = 7053)	Male (n = 4356)	Female (n = 2960)	*P*
Corrected Ca patients, n	5583	3329	2254	
Corrected Ca (mmol/L)	2.21 (2.05, 2.37)	2.19 (2.03, 2.34)	2.24 (2.08, 2.40)	< 0.001
**Corrected Ca, n (%)**				
< 2.1	1747 (31.3)	1131 (34.0)	616 (27.3)	< 0.001
2.1–2.5	3214 (57.6)	1898 (57.0)	1316 (58.4)	0.309
> 2.5	622 (11.1)	300 (9.0)	322 (14.3)	< 0.001
P patients, n	6413	3806	2607	
P (mmol/L)	1.78 (1.40, 2.18)	1.79 (1.41, 2.21)	1.75 (1.37, 2.14)	0.003
**P, n (%)**				
< 0.87	260 (4.1)	150 (3.9)	110 (4.2)	0.579
0.87–1.45	1561 (24.3)	901 (23.7)	660 (25.3)	0.132
1.46–1.78	1423 (22.2)	833 (21.9)	590 (22.6)	0.481
> 1.78	3169 (49.4)	1922 (50.5)	1247 (47.8)	0.036
iPTH patients, n	5610	3355	2255	
iPTH (pg/mL)	314.3 (164.0, 560.0)	317.0 (173.0, 563.6)	308.2 (149.7, 555.0)	0.132
**iPTH, n (%)**				
< 150	1249 (22.3)	685 (20.4)	564 (25.0)	< 0.001
150–300	1448 (25.8)	912 (27.2)	536 (23.8)	0.004
301–600	1639 (29.2)	982 (29.3)	657 (29.1)	0.914
601–800	439 (7.8)	262 (7.8)	177 (7.8)	0.956
> 800	835 (14.9)	514 (15.3)	321 (14.2)	0.263

Serum P (29.0% *vs.* 22.4% *vs.* 19.9%, *P* < 0.001) and iPTH (57.6% *vs.* 53.5% *vs.* 53.3%, *P* < 0.001) among patients aged > 60 years were higher than those of other groups. Further, percentages of subjects with hyperphosphatemia (65.5% *vs.* 74.3% *vs.* 77.2%, *P* < 0.001) and hyperparathyroidism (16.4% *vs.* 36.1% *vs.* 43.8%, *P* < 0.001) were reduced among subjects aged > 60 years *vs.* other those of other age groups. Further, the incidence of hypoparathyroidemia (25.9% *vs.* 21.9% *vs.* 16.6%, *P* < 0.001) was increased in the elderly *vs.* other age groups. Corrected Ca level differences between groups were not statistically significant (*P* > 0.05) (Table 5).

**Table 5:** The levels of Ca, P, and iPTH in different-age groups.

	All patients (n = 7053)	18–44 (n = 1414)	45–59 (n = 3016)	≥ 60 (n = 2623)	P
Corrected Ca patients, n	5583	1049	2388	2146	
Corrected Ca (mmol/L)	2.21 (2.05, 2.37)	2.22 (2.06, 2.39)	2.21 (2.06, 2.37)	2.20 (2.05, 2.36)	0.041
**Corrected Ca, n (%)**					
< 2.1	1747 (31.3)	308 (29.4)	738 (30.9)	701 (32.7)	0.145
2.1–2.5	3214 (57.6)	611 (58.2)	1379 (57.7)	1224 (57.0)	0.788
> 2.5	622 (11.1)	130 (12.4)	271 (11.3)	221 (10.3)	0.192
P patients, n	6413	1279	2747	2387	
P (mmol/L)	1.78 (1.40, 2.18)	1.89 (1.49, 2.30)	1.83 (1.44, 2.22)	1.67 (1.32, 2.07)	< 0.001
**P, n (%)**					
< 0.87	260 (4.1)	37 (2.9)	92 (3.3)	131 (5.5)^a^	< 0.001
0.87–1.45	1561 (24.3)	255 (19.9)	614 (22.4)	692 (29.0)^a^	< 0.001
1.46–1.78	1423 (22.2)	257 (20.1)	599 (21.8)	567 (23.8)^b^	0.032
> 1.78	3169 (49.4)	730 (57.1)	1442 (52.5)	997 (41.8)^a^	< 0.001
iPTH patients, n	5610	1154	2405	2051	
iPTH (pg/mL)	314.3 (164.0, 560.0)	400.7 (217.9, 681.0)	314.3 (164.0, 560.0)	266.1 (146.1, 463.1)	< 0.001
**iPTH, n (%)**					
< 150	1249 (22.3)	191 (16.6)	527 (21.9)	531 (25.9)^a^	< 0.001
150–300	1448 (25.8)	222 (19.2)	612 (25.4)	614 (29.9)^a^	< 0.001
301–600	1639 (29.2)	394 (34.1)^c^	676 (28.1)	569 (27.7)	< 0.001
601–800	439 (7.8)	111 (9.6)	192 (8.0)	136 (6.6)^b^	0.010
> 800	835 (14.9)	236 (20.5)	398 (16.5)	201 (9.8)^a^	< 0.001

^a^Patients over 60 years old compared with the other two age groups, *P*-int < 0.05.

^b^Patients over 60 years old compared with patients under 45 years old, *P*-int < 0.05.

^c^Patients under 45 years old compared with the other two age groups, *P*-int < 0.05.

## Discussion

CKD-MBD is prevalent in CKD patients, especially in dialysis patients. One of the important steps in managing CKD-MBD is to understand the current management status of CKD-MBD. DOPPS5 only reports results for some of the richer regions in China, but there are many regions in China with relatively low economic levels. In order to understand the level of CKD-MBD management across China, we combined questionnaire with real-world survey. In the questionnaire, we compared Sichuan, located in western China, with the eastern region; in the real-world study, we compared the findings with DOPPS5.

iPTH control is no doubt an essential part of CKD-MBD management. The risk of all-cause and cardiovascular mortality increases with iPTH. Risk of all-cause mortality increases by 23% when iPTH levels are > 600 pg/mL and mortality risk is lowest when iPTH is 150–300 pg/mL^12^. In the questionnaire, although iPTH achievement rate was nearly 50% when a target range of 150–600 pg/mL was set. However, that of iPTH was only 33.8% when a target range of 150–300 pg/mL was set. It shows that there is still huge room for improvement in the iPTH achievement rate. Increasing the frequency of detection can improve the achievement rate by which patients achieve target iPTH concentrations^13^. However, the result revealed that 25.0% of centers did not pay attention to iPTH examinations. They checked iPTH levels every six months or failed to perform evaluations of iPTH, which affected SHPT control. The improvement of iPTH management should include the strengthening of the implementation of increased iPTH level assessment.

The use rates of prescribed medications can also affect achievement rate. From the questionnaire, we can see that oral calcitriol was the primary SHPT therapy in China. The use rates of intravenous vitamin D and vitamin D receptor activator preparation were minimal, and over 80% of centers had use rates < 10%. Many scholars recommend calcimimetics, especially cinacalcet, to reduce levels of iPTH. DOPPS5 showed that the use of cinacalcet in European and American countries was 13% to 34%, and that in Japan was 24%. In comparison, the cinacalcet utilization rate in China was only 1%^11^. In this study, the use rate of calcimimetics for more than 50% of centers was less than 10%.

In the questionnaire, the achievement rate of P was only 26.7% and nearly 60% of centers had achievement rates less than 30%. Because medication use rates are closely related to achievement rates, we similarly investigated the use of P-lowering medications. The utilization of Ca-containing P binders was less than 50% in than 60% of centers. This finding reflected the popularization of new P-reducing treatment methods. With regard to the use of non-Ca-P binders, only 21.3% (58/272) centers had binder utilization rates of < 10%, while 33.8% (92/272) centers had a binder utilization rate of 11–30%. The frequency of the application of non-Ca-containing phosphate binders was significantly greater than that of intravenous calcitriol and oral calcimimetic. It reflects the tradition of acknowledging the importance of phosphate management in our country. It is also associated with the introduction of the clinical use of non-Ca-containing phosphate binders. It can also be seen from the questionnaire that the use of aluminum-containing phosphate binders was deficient. As a salvage method for treating patients with extremely high P levels, 90.4% of centers had an occupancy rate of < 10%.

Achievement rates may be influenced by economic factors such as GDP and healthcare resources. As we all know, the eastern region basically belongs to the coastal cities and is the most economically developed region in China, while Sichuan belongs to the western region, and there is a big difference in economic level between the two regions. Thus, we compared Sichuan with the eastern region in the questionnaire. Results showed that both iPTH and serum Ca achievement rates were lower in Sichuan compared to the eastern region of China. However, there was no significant difference in serum P achievement rate between the two regions.

Different Ca concentrations in dialysis solutions can affect Ca and P metabolism in HD patients. The use of low-Ca dialysate helps reduce the risk of hypercalcemia. The results showed that 87.9% of centers routinely used 1.5 mmol/L Ca dialysate, and about 1/3 of centers routinely used 1.25 mmol/L Ca dialysate. Use of 1.75 mmol/L Ca dialysate is not common. Gradually decreasing Ca dialysate concentrations is a global trend.

The questionnaire is subjective. To further clarify the current management status of MBD, we extracted data from the Sichuan province data system to perform real-world research separately. On the one hand, we compared the study results with DOPPS5; on the other hand, we analyzed the achievement rate stratified by age, gender, and hospital level. We found that nearly half of all patients considered had corrected Ca and iPTH levels within target ranges. Only 25.5% of patients had P levels within the target range defined as 0.81–1.45 mmol/L, a finding similar to that of the questionnaire.

Data from the real-world of Sichuan showed that 25.8% of patients achieved 150-300 pg/mL, a percentage lower than of Europe and North America^11^. When the range was set to 150–600 pg/mL, 55.0% of patients achieved target. The achievement rate remained lower than that of North America. A large gap between the achievement rate of Sichuan in the real-world and the nearly 100% achievement rate of Japan was also observed. Here, patients with hyperparathyroidism accounted for 22.7% of the study population. Patients with iPTH levels exceeding 800 pg/mL accounted for 14.9%; however, according to DOPPS5, only 1% Japanese HD patients had iPTH exceeding 600 pg/mL^11^. There are some reasons why iPTH management in Sichuan in the real-world is far from Japan. We carefully analyzed the possibility that the difference was related to Japan's narrow target range (60–240 pg/mL) and its higher detection frequency^14^. DOPPS5 showed that the detection frequency in Japan was high, and the monthly detection rate of iPTH in their patients reached 23.0%. In comparison, the monthly detection rate of iPTH in China was only 3.2%^11^.

It was observed that the older the individual, the lower the iPTH level, and the easier it was to achieve standard iPTH levels. Further, the incidence of hypoparathyroidemia in patients aged > 60 years was significantly higher than that in young people. A previous study reported that age was associated with a reduction in iPTH level^15^. Patients with low iPTH levels are prone to complications including low transport bone disease or even akinetic bone disease. This is because those with low iPTH levels are prone to bone turnover and formation slowing, which reduces the buffering capacity of circulating Ca and P. In this case, it is easy to cause arterial calcification and death^16,17^. An increased incidence of low transport bone disease was observed in elderly patients. HD patients with low iPTH levels had higher rates of mortality and non-fatal cardiovascular events^18^. This finding reminded us that physicians should not only pay attention to the achievement rate of iPTH but also the occurrence of hypoparathyroidemia in elderly patients.

For every 1 mg/dL increase in P, all-cause mortality increases by 18%, and cardiovascular mortality increases by 10%^19^. Controlling levels of P can reduce mortality. In the real-world survey, the achievement rate of P levels within the target range in Sichuan was determined to be far lower than those of Europe, North America, Japan, and China when DOPPS5 data were assessed^11^. P levels > 1.45 mmol/L were observed in 71.6% of patients. In fact, 49.4% of patients had P levels > 1.78 mmol/L.

These findings indicate that phosphate management is quite an issue. Lack of knowledge of hyperphosphatemia is one reason for which phosphate management is insufficient. Low literacy levels and communication barriers with physicians also negatively impact treatment. Many ethnic minorities in Sichuan do not speak Mandarin. Phosphate is easily affected by food. Most foods contain P mainly processed products such as sausages, bacon, and hot pot. Compliance is another critical factor that affects P levels. Patient non-compliance increases as the number of phosphate binder pills taken daily increases^20^. Simplifying the medication regimen may increase patient compliance and improve the overall P levels. Toussaint et al.^21^ showed that intensive education has the potential to significantly improve CKD-MBD control and the achievement of target P levels. Therefore, additional patient education strategies should be developed.

Target phosphate level achievement rates of tertiary hospitals were better than those of secondary and lower hospitals in the real-world survey. Further, patients in secondary and lower hospitals were at significantly increased risk of hyperphosphatemia. Secondary and lower *vs.* tertiary hospital-related differences may be associated with different management strategies, medical staff guideline implementation, and medical conditions of both types of hospitals. Improved rates of reaching phosphate target levels and a reduced incidence of hyperphosphatemia were noticed among elderly patients. This finding may be attributed to low levels of food intake and excellent adherence to phosphate management observed among patients on long-term dialysis. Older patients tend to better comply with treatment than younger patients^22^. However, some studies have shown that compared with young patients, older patients have increased rates of mortality^23,24^.

DOPPS1 ~ 3 showed that mortality risk is the lowest when Ca levels are within the standard range. Both hypercalcemia and hypocalcemia increase all-cause mortality risk^7^. In the real-world survey, 57.6% of patients had Ca levels within the standard range, while 31.3% suffered from hypocalcemia, a rate higher than that of the DOPPS5 in China (20.9%), Europe (20%), and North America (12%)^11^. However, hypercalcemia dramatically improved when compared with a multicenter study conducted by Zhang L et al.^25^. Consistent with the trend of continuous improvement in HD management, the use of Ca-containing P binders is gradually decreasing since calcimimetics control iPTH levels better than other drugs in MHD patients. This reduces the Ca elevation risk.

Comprehensive compliance to MBD biomarker target levels can reduce the risk of all-cause mortality^26^. A previous study showed that compared with the comprehensive achievement of target levels of above three MBD parameters, mortality risk increased by 51% in patients who didn’t achieve all three targets. Further, the risk of those with levels outside any two and a single target range increased 35–39% and 15–21%, respectively^9^. The comprehensive achievement rate in Sichuan in the real-world study was only 7.5%, a value lower than that of other studies^8,27,28^. Improvement of comprehensive management strategies is of the utmost importance.

This cross-sectional, multicenter study included data from centers within the west, central, and east of China. To a certain extent, this study represents the current status and diversity of hemodialysis management in China. Real-world survey data collected via the hemodialysis data platform was authentic and accurate, and accurately reflects MBD management. Our study has certain limitations. Factors such as vintage, medications, infection, and tumor may affect the achievement rates. We failed to discuss the influence of the above factors on the achievement rates in the real-world survey.

## Conclusion

Achievement rates of MBD parameters were low among the study population, especially with regard to serum P. The comprehensive achievement rate is very low. Phosphate management in secondary and lower hospitals and young dialysis patients should be strengthened.

## Data Availability

The data used of this study are available from the corresponding author on reasonable request.
